# Multi-disciplinary community respiratory team management of patients with chronic respiratory illness during the COVID-19 pandemic

**DOI:** 10.1038/s41533-022-00290-y

**Published:** 2022-08-13

**Authors:** Emily Turner, Emma Johnson, Kate Levin, Stewart Gingles, Elaine Mackay, Claire Roux, Marianne Milligan, Marion Mackie, Kirsten Farrell, Kirsty Murray, Suzanne Adams, Joan Brand, David Anderson, Hannah Bayes

**Affiliations:** 1 Respiratory Medicine, NHS Greater Glasgow and Clyde, Glasgow, UK; 2 Public Health Directorate, NHS Greater Glasgow and Clyde, Glasgow, UK; 3 Community Respiratory Response Team, NHS Greater Glasgow and Clyde, Glasgow, UK

**Keywords:** Outcomes research, Health services, Palliative care

## Abstract

The Greater Glasgow & Clyde NHS Trust Community Respiratory Response Team was established to manage patients with chronic respiratory disease at home during the COVID-19 pandemic. The team aimed to avert hospital admission while maximally utilising remote consultations. This observational study analysed outcomes of the triage pathway used, use of remote consultations, hospital admissions and mortality among patients managed by the team. Patients’ electronic health records were retrospectively reviewed. Rates of emergency department attendance, hospital admission and death within 28 days of referral were compared across triage pathways. Segmented linear regression was carried out for emergency admissions in Greater Glasgow and Clyde pre- and post- Community Respiratory Response Team implementation, using emergency admissions for chronic obstructive pulmonary disease in the rest of Scotland as control and adjusting for all-cause emergency admissions. The triage category correlated with hospital admission and death. The red pathway had the highest proportion attending the emergency department (21%), significantly higher than the amber and green pathways (*p* = 0.03 and *p* = 0.004, respectively). The highest number of deaths were in the blue “end-of-life” pathway (*p* < 0.001). 87% of interactions were undertaken remotely. Triage severity appropriately led to targeted home visits. No nosocomial COVID-19 infections occurred among patients or staff. The Community Respiratory Response Team was associated with a significant decrease in emergency admissions (RR = 0.96 for each additional month under the Poisson model) compared to the counterfactual if the service had not been in place, suggesting a benefit in reducing secondary care pressures. The Community Respiratory Response Team effectively managed patients with chronic respiratory disease in the community, with an associated reduction in secondary care pressures during the COVID-19 pandemic.

## Introduction

The COVID-19 pandemic resulted in significant pressure on healthcare services worldwide. Services were re-designed to manage existing healthcare needs alongside the burden of COVID-19-associated illness^1^. In the UK, a significant volume of elective healthcare activity was postponed to deal with rising COVID-19 cases. Between April 2020 and July 2021, the BMA estimated there were over 26 million fewer NHS outpatient attendances compared with the previous years^2^ and, in Scotland, 396,771 patients still awaited new outpatient appointments as of August 2021^3^. The burden the pandemic placed on secondary care resources, as well as the increased risks of COVID-19 infection in patients with chronic health conditions, created a need to manage patients away from the hospital setting, particularly patients with pre-existing respiratory diseases.

Delivering effective care in the community was a key focus of the Scottish response to COVID-19^4^, and the redeployment of healthcare staff to support community services during the pandemic provided the opportunity to assess the utility of multidisciplinary community care.

NHS Greater Glasgow and Clyde (GGC) is the largest health board in the UK, with a catchment population of 1.14 million across both urban and rural settings^5^. Within the Trust, there is a high burden of chronic respiratory disease with an overall prevalence of COPD in those aged over 45 years of 6.26%^6^. About 34,115 hospital bed days were attributed to COPD in NHS GGC in 2018/2019, the highest for any trust in Scotland^7^.

The NHS GGC Community Respiratory Response Team (CRRT) was created in March 2020 as an emergency measure to support patients with chronic respiratory illnesses in their own homes during the COVID-19 pandemic. Its aim was to avert hospital admission, where possible, in order to reduce the risk of nosocomial COVID-19 infection and to alleviate pressure on acute medical services. The service operated from 31 March to 30 Sept 2020 for the first UK COVID-19 wave and reformed for the second wave in November 2020.

The CRRT was formed by amalgamating six community respiratory teams across NHS GGC to create a multidisciplinary team (MDT) led by respiratory specialist nurses, physiotherapists, occupational therapists and pharmacists, with remote respiratory consultant support. The team operated between 8 a.m. and 6 p.m. 7 days a week throughout the pandemic and accept referrals for all respiratory diagnoses from all patients in NHS GGC. It received over 1200 referrals and provided over 3600 consultations, including both telephone consultations and home visits to patients, in its first 66 days; averaging 20 referrals and 70 consultations per day.

**Fig. 1:**
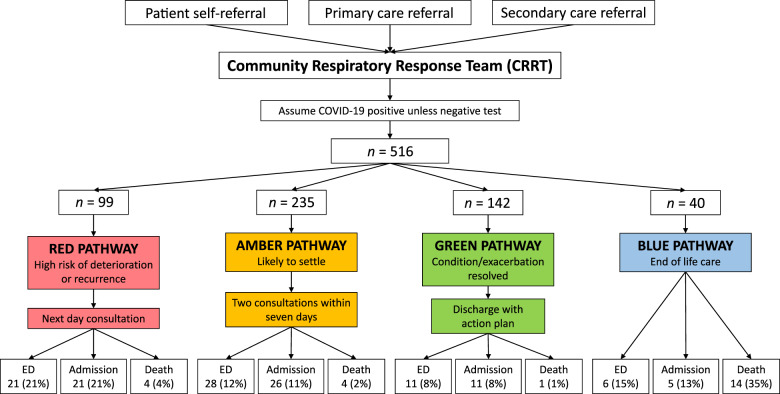
CRRT triage pathway and patient outcomes by pathway. Outcomes of patients referred to the CRRT. Percentage values refer to proportions of individual pathways.

A referral was made electronically by primary care, COVID assessment centre staff, secondary care, or patient self-referral. The majority of patients received a telephone contact from the service on the day of referral. Patients who had been discharged from the hospital or begun treatment via their GP on the day of referral were contacted the following day. Senior clinical team members, defined as advanced nurse practitioners (NHS Scotland Band Seven) or senior nurses (NHS Scotland Band Six) experienced in community respiratory care, made this initial contact and triaged patients into “red”, “amber” or “green” pathways denoting high to low risk of imminent admission; or as “blue” for patients who required home oxygen or other respiratory intervention as part of the end-of-life care (Fig. 1). Triage decisions were made clinically based on the review of electronic patient records, referral details and information provided by the patient regarding whether they felt their condition continued to worsen, had stabilised or was improving.

Patients triaged as red received a further consultation, either at home or via telephone, within 24 h. Patients triaged as amber received two contacts within 1 week. Patients triaged as green were discharged from the service with a management plan. Patients triaged as blue were provided with equipment or palliative input as required. Remote reviews were undertaken where possible, with home visits minimised and personal protective equipment (PPE) utilised to minimise the risk of COVID-19 infection to patients and staff. Daily virtual ward rounds were undertaken to discuss patients of clinical concern with senior team members.

Previous studies have shown that multidisciplinary and nurse-led community respiratory teams are effective and can lead to a reduction in emergency hospital admissions among respiratory patients^8–12^. This study analysed the effectiveness of the CRRT service overall by assessing the key components and outcomes of the CRRT triage pathway in prioritising patients for review, use of remote patient-clinician interactions, and the impact of the service on hospital attendance and mortality.

The primary aims were to determine (1) whether the triage system was effective in identifying high-risk patients; (2) how extensively remote consultations were used and face-to-face consultations targeted towards patients most in need; and (3) whether CRRT input affected rates of emergency department attendances (EAs), hospital admission and death among patients at 28 days after an initial consultation.

## Methods

### Study population and data sources

This was a population-based, retrospective, observational study. Data were collected from the electronic records of all patients referred to the CRRT during the month of May 2020. Data on the reason for referral, triage category, primary respiratory diagnosis and patient demographics were collected from the original referral documentation to the CRRT. Information on the number and type of consultations was collected from the CRRT electronic consultation notes. Data on admissions and deaths within 28 days of referral to CRRT were collected from hospital admission records and, where further information was necessary, from inpatient notes and discharge letters viewable in the electronic patient record. The six teams provided the total numbers of staff involved, percentage of their time committed to the CRRT, and the number of staff working per day. These were used in conjunction with payroll details to calculate the cost of staffing the service. Monthly emergency attendance (EA) data for all causes and COPD only (COPD EA) from January 2018 to May 2021 were obtained for NHS GGC and the rest of Scotland, with the exception of Fife, Lothian and Tayside health boards, from Public Health Scotland. COPD EAs are defined as those coded as ICD-10 J40-J44^13^.

### Statistical analysis

Two stages of analysis were undertaken. The first stage analysed individual-level data to produce data summaries and compare the utility of the service in its various forms and outcomes between triage pathways. Chi-squared tests were used for comparisons of pathways.

The second stage used ecological data to measure the impact of the service on emergency hospital attendance. Using regional data, emergency department (ED) attendances with a primary diagnosis of COPD in GGC was compared before and after the onset of the service with a 1-month phase-in period, using segmented linear regression. The rest of Scotland (apart from Fife, Tayside and Lothian) (RoS) was used as a control. The model adjusted for all-cause EAs to control for the impact of COVID-19 on COPD EAs during this period. The model was carried out first using linear regression and included autoregressive terms^14,15^ and a Fourier term to adjust for seasonality^16^. A Poisson model was then run—preferable for count data—also with a Fourier term adjustment. Sensitivity analyses included removing the final data point and significant outliers and modelling with and without adjustment for autoregression, as recommended^17^. Poisson models also included adjusting for all-cause EAs as an offset term, i.e. adjustment for the rate of COPD EA per all-cause EA. These models are available on request from the authors. These models produced different parameter estimates but the same overall conclusion.

### Ethics

Both the patient and public health Scotland data used were collected as part of service evaluation and therefore did not require research ethics committee approval^18^. For the same reason, written informed patient consent was not sought.

### Reporting summary

Further information on research design is available in the Nature Research Reporting Summary linked to this article.

## Results

### Patient demographics

About 516 patients were referred to the CRRT in May 2020. All patients were residents within the NHS GGC health board area. COPD was the commonest primary diagnosis comprising 65% of patients referred, and this increased to 72% when COPD/asthma overlap patients were included. The second most common diagnosis was asthma (12%), followed by interstitial lung disease (ILD) (7%), with smaller numbers of patients with other respiratory diagnoses (Table 1). Diagnoses classed as “other” included obstructive sleep apnoea, pulmonary embolism, and lung cancer. The mean age of patients referred was 69. 340 (66%) patients were female and 176 (34%) were male. Females outnumbered males in all primary respiratory diagnoses with the exception of ILD, in which 21 of the 36 patients were male (Table 1).

**Table 1:** Patient demographics *n* = number and overlap refers to a primary respiratory condition with features of both asthma and COPD.

Primary Respiratory Condition	*n*		Mean age (Median, IQR)	Female:Male
Total	516		69 (71, 62–79)	1.93
COPD	336	65%	71 (72, 64–79)	1.87
Asthma	63	12%	51 (52, 43–60)	3.85
ILD	36	7%	75 (78, 68–82)	0.71
Overlap	34	7%	71 (71, 62–79)	1.43
COVID-19	11	2%	74 (88, 82–91)	1.75
Bronchiectasis	10	2%	84 (73, 68–76)	9.00
End-of-life care	9	2%	82 (80, 75–91)	3.50
Other/Unknown	17	3%	71 (70, 64–83)	3.25

### ED attendance, hospital admissions, and death rates

Sixty-six patients (13%) attended ED within 28 days of referral to the CRRT. Fifty-five patients (83%) who attended ED were subsequently admitted to the hospital, suggesting a genuine need for inpatient care among the majority of patients who attended ED. Eight patients were admitted directly to the hospital without attending ED, meaning a total of 63 patients (12%) were admitted to the hospital (Table 2 and Supplementary Table 1).

**Table 2:** Patient Demographics and Outcomes by CRRT Triage Pathway % relates to proportion of pathway.

Pathway	*n*	Gender		Mean Age (Median, IQR)	ED Attendance		Admitted		Died	
		F	M			%		%		%
RED	99	67	32	71 (72, 62−78)	21	21%	21	21%	4	4%
AMBER	235	154	81	68 (70, 60−78)	28	12%	26	11%	4	2%
GREEN	142	89	53	68 (70, (61−78)	11	8%	11	8%	1	1%
BLUE	40	30	10	81 (84, 75−87)	6	15%	5	13%	14	35%
TOTAL	516	340	176	69 (71, 62−79)	66	13%	63	12%	23	4%

Twenty-five patients (4%) died within 28 days of referral. The mean number of days from referral to death was 11 (range 0–28, median 12, IQR 3–15). The mean age at death was 78 years (range 53–98, median 79, IQR 72–84). Fifteen patients who died were female and ten were male. Eight (32%) patients who died within 28 days were nursing home residents. Deaths occurred in 11 patients with COPD (3% of COPD patients), one patient with COPD/asthma overlap (3% of overlap patients), one patient with bronchiectasis (10% of the group), and five patients identified as end-of-life (56%). Two patients died in the other/unknown group (12%), both of whom had metastatic malignancies. Six patients died after a COVID-19 diagnosis, three of whom were triaged with COVID-19 as the primary diagnosis, and five patients had confirmatory virology testing. No deaths occurred in patients with a primary respiratory diagnosis of asthma.

### Utility of the triage pathway

19% of patients were triaged as red, 46% as amber, 27% as green and 8% as blue (Fig. 1). Scoring the red, amber and green pathways according to triage severity (where red is highest and green is the lowest severity), attendance, admission and death correlated negatively (R = −0.98, −0.96 and −0.97 respectively), i.e. the higher the triage severity, the smaller the proportion attending or being admitted.

The red pathway had the highest proportion attending ED (21%) and this was significantly higher than the amber and green pathways (*p* = 0.03 and *p* = 0.004, respectively), but not the blue pathway (*p* = 0.419). As only seven patients were admitted directly to the hospital without attending ED, the proportions of the 63 patients admitted were similar—21 (21%) red, 26 (11%) amber, 11 (8%) green and five (13%) blue pathway patients (Table 2). The highest number of deaths were in the blue “end-of-life” triage pathway (*P* < 0.001 when compared with all other pathways), followed by the red “high risk of deterioration” pathway (Table 2). The one patient who passed away in the green triage pathway died from acute abdominal sepsis, meaning no patients triaged as green subsequently died from a respiratory cause.

### Utilisation of remote consultations

The total number of CRRT consultations in the study population was 2261. Of these, 1971 (87%) were conducted remotely via telephone or an online video calling platform. For their initial consultation, 431 (84%) patients were assessed by remote consultation and 85 (16%) at a home visit. About 181 patients (35%) had at least one home visit during follow-up (mean 1.6; range 1–8; median 1.6, IQR 1–2), meaning 335 patients referred (65%) were managed entirely remotely.

Patients received, on average, 4.4 consultations (range 1–44; median 3; IQR 1–5). A higher number of consultations was not significantly associated with ED attendance (Pearson’s correlation *r* = 0.036, *p* = 0.416), hospital admission (*r* = 0.074, *p* = 0.095) or death (*r* = −0.085, 0.055). This was also true when the blue pathway data were removed from the analysis. 33% of patients who presented to ED had only one CRRT consultation, while just 22 (13%) of the 169 patients who had only one consultation attended ED.

The proportion of patients receiving a home visit correlated with triage category severity. A significantly greater proportion received a home visit in the red pathway; 71% of patients compared with 32% of amber patients (*p* < 0.001), 18% of green patients (*p* < 0.001) and 28% of blue patients (*p* < 0.001). Red pathway patients were also more likely to receive a home visit as their first consultation, with 30% of red pathway patients receiving a face-to-face review at home, compared with 12% of amber (*p* < 0.001), 15% of green (*p* = 0.005) and 13% of blue patients (*p* = 0.037). Of note, a large number of blue pathway patient referrals were requests for palliative oxygen delivery rather than requests for patient review; this includes ten (71%) of the 14 blue pathway patients who died and 38% of all patients who died within 28 days of referral.

Patients seen face-to-face initially after referral were not significantly more likely to attend ED (15% of initial home visits vs 12% of remote consultations, *p* = 0.388), be admitted to hospital (15 vs 11%, *p* = 0.288), or die within 28 days (5 vs 4%, *p* = 0.802).

Importantly, no nosocomial COVID-19 infections were identified due to CRRT input among CRRT staff or patients managed at home.

### Staffing and cost analysis

In total, the CRRT employed a total of 26 nurses, 12 physiotherapists, one occupational therapist and two respiratory consultants. Of the staff employed, 25 worked full time for the CRRT while the others either worked part-time or combined work for the CRRT with other duties. 93% of referrals were made on weekdays, with an average of 24 referrals per weekday and 4 per weekend day. Staffing was adjusted accordingly, with an average of 23 staff working each weekday and 13 each weekend to cover new referrals and follow-up consultations. The team provided an average of 490 consultations per week, meaning each staff member averaged 14 consultations per day, including telephone consultations and home visits. Respiratory consultant input to the daily virtual ward rounds required approximately three sessions per week with an annual cost estimated at £28,000 per annum.

Based on the above staffing levels, the cost of staffing the service across the NHS GGC was approximately £48,789 per week, or £2,543,815 per annum (Table 3). Therefore, the average cost was £86 per CRRT consultation and £378 per patient referred in May 2020. This is compared to an average cost of £3602 per patient for a secondary care admission in NHS GGC in 2018–19^19^ and £3000 per COPD-related inpatient stay in NHS Scotland^20^.

**Table 3:** Projected costs of staffing the CRRT based on staffing levels required during the month of May 2020.

	WTE	Hours	£	£
Band 7 specialist nurse	6.8	255	9306	485,237
Band 6 nurse	20.8	780	24,155	1,259,442
Band 7 physiotherapist	3	112.5	4106	214,075
Band 6 physiotherapist	8.4	315	9755	508,621
Band 6 occupational therapist	0.8	30	929	48,440
Respiratory consultant	3.3	12	538	28,000
	43.1	1504.5	48,789 per week	2,543,815 per annum

### Effect on COPD ED attendances

**Fig. 2:**
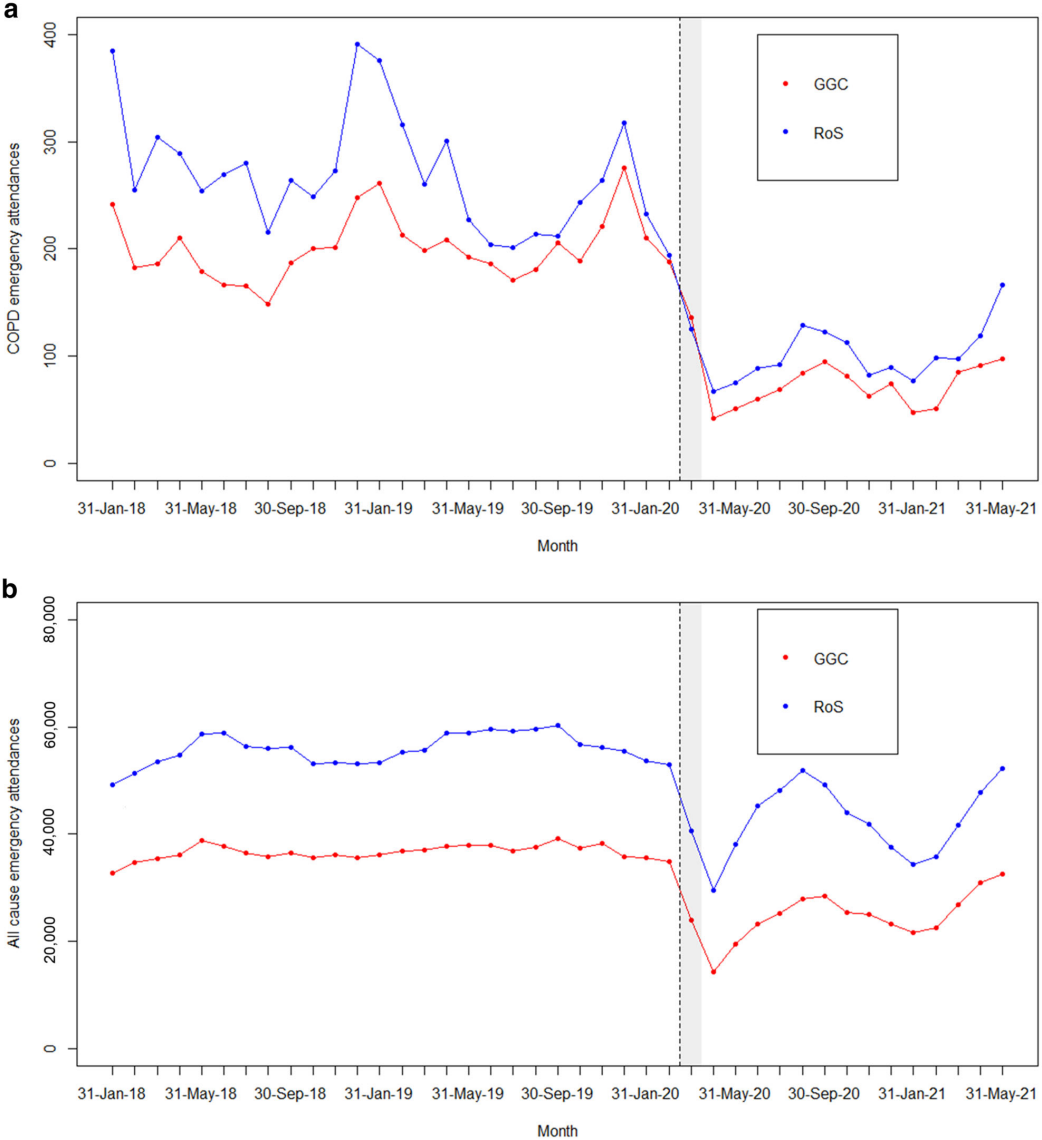
COPD and all-cause emergency attendances (EAs) per month, January 2018–May 2021, in Greater Glasgow and Clyde (GGC) and the Rest of Scotland, excluding Fife, Lothian and Tayside (RoS). **a** COPD EAs per month, for residents of GGC and RoS. **b** All-cause EAs per month for residents of GGC and RoS. The shaded area represents the phase-in period of the community respiratory service.

**Fig. 3:**
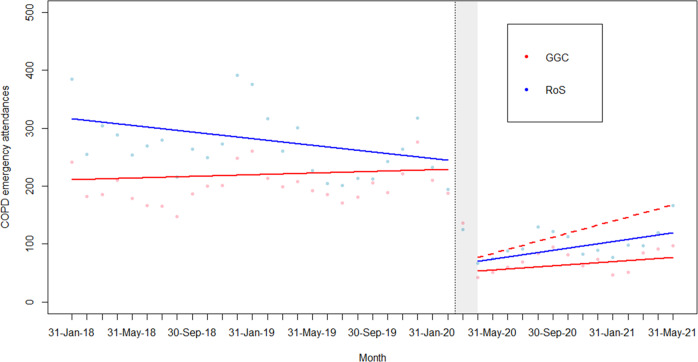
The effects of the CRRT on COPD emergency admissions. Segmented linear regressions of COPD EAs in Greater Glasgow and Clyde with the Rest of Scotland, excluding Fife, Lothian and Tayside (RoS) as control and adjusting for all other cause emergency attendances (EAs), January 2018–May 2021; deseasonalized linear trend under model and the predicted trend had the community respiratory project not been implemented.

Patients with COPD were the most represented group referred to the CRRT (Table 1 and Supplementary Table 1). Prior to implementation of the CRRT, increases in COPD EAs over time were evident in GGC, while the trend for all-cause EAs was approximately flat (Fig. 2). Conversely, in RoS, COPD EAs were decreasing, and all-cause EAs saw a slight rise over time. Following the start of the pandemic and the establishment of the CRRT service, a large drop was seen in both GGC and RoS in all-cause EAs and COPD EAs. This was then followed by a sharp increase, reduction and second increase in all-cause EAs (likely reflecting the waves of the pandemic as people attended ED less during lockdown periods). COPD EAs did not, however, see as large an increase in either area (Fig. 2). Adjusting for changes in COPD EAs in RoS, there is a significant decrease in the trend in COPD EAs in GGC (RR = 0.96 (0.94, 0.98) for each additional month under the Poisson model) compared with the counterfactual, i.e. if the service had not been in place (Fig. 3 and Table 4).

**Table 4:** Parameter estimates, standard error, relative risk and 95% confidence intervals, and *p* values from the segmented Poisson regression predicting COPD EAs, adjusting for seasonality.

	Parameter estimate	SE	RR (95% CI)	p-value
Intercept	3.402	0.223	30.02 (19.40, 46.46)	<0.001
Additional GGC level at baseline	0.302	0.083	1.35 (1.15, 1,59)	<0.001
Trend pre-intervention	0.020	0.002	0.98 (0.98, 0.98)	<0.001
Additional GGC trend pre-intervention	0.018	0.002	1.02 (1.01, 1.02)	<0.001
All other cause of EA trend (per 1000)	0.044	0.004	1.04 (1.04, 1.05)	<0.001
Level change post-phase-in period	−0.166	0.100	0.85 (0.70, 1.03)	0.099
Trend change post-phase-in period	0.025	0.007	1.03 (1.01, 1.04)	<0.001
Additional level change in GGC	−0.191	0.103	0.83 (0.68, 1.01)	0.063
Additional trend change in GGC	−0.039	0.011	0.96 (0.94, 0.98)	<0.001

## Discussion

The NHS GGC CRRT was established during the COVID-19 pandemic to provide multidisciplinary care for patients with chronic respiratory illnesses in their own homes. Our observational data demonstrates that the CRRT was able to efficiently manage a large number of patients, utilising extensively remote consultants, and with low levels of admission to secondary care.

Triage category correlated with a higher risk of hospital attendance and death and appropriately targeted home visits by the CRRT. This demonstrates that the CRRT triage pathway was able to effectively identify patients at risk of deterioration and provide them with appropriate support in the community. The only death among patients triaged as green was from an unexpected, non-respiratory cause and there were no deaths among asthma patients, a group, particularly at risk of rapid decline for whom correct triage and timely referral to hospital is particularly important. Therefore, we conclude that the triage pathway allowed for efficient assessment of the likelihood of deterioration, with low rates of mortality and hospital admissions seen within the CRRT cohort.

Respiratory patients are at increased risk of hospital-acquired infection^21^, poorer outcomes following COVID-19^22^ and are vulnerable to other community-acquired infections, such as a respiratory syncytial virus (RSV)^23^. With the caveat that widespread testing for COVID-19 was not available at the beginning of the pandemic, there were no known nosocomial COVID-19 infections among CRRT staff or patients, meaning the service potentially averted healthcare-acquired infections in this vulnerable patient group.

The CRRT used predominantly remote consultations and our data shows that the triage system appropriately targeted time-intensive strategies such as home visits to the most unwell patients. Previous studies of telemedicine in COPD have had mixed results with no clear benefit identified^24–27^. This study demonstrates the effective application of telemedicine backed up by face-to-face consultations to provide cost-effective care while alleviating concerns about safety from purely virtual consultations^28^. Triage decisions were based on the judgement of experienced clinicians in discussion with patients, with limited use of home pulse oximetry. We are therefore unable to comment on predictive factors for triage severity and 28-day outcomes. However, the use of predictive models and artificial intelligence to predict patient outcomes in COPD is a promising area^29,30^ which could be explored through an ongoing prospective analysis of the service.

Hospitalisations for patients with COPD have been higher in GGC than in Scotland as a whole for the last two decades and this gap has been widening^31^. This may be due to a reduction in incidence at the older ages among males, the benefit of which would be felt largely outwith GGC^32^, which is home to a younger and more socioeconomically deprived population^6^. It may also be linked with geographical differences in severity of disease and comorbidity, as well as inter-centre differences in investigation and treatment, previously shown to be associated with outcomes for lung cancer^33^. Emergency attendance rates were low among patients seen by the CRRT and CRRT input was associated with a significant reduction in the number and trend of COPD EAs in the Trust. Factors related to the national lockdown, including fear of attending hospital, lower risk of community-acquired infection and reductions in air pollution, likely contributed to reduced admissions across Scotland. However, when adjusted for national trends, EAs in GGC still dropped following the initiation of the CRRT, suggesting the team did achieve its aim of alleviating pressure on acute medical services.

The most significant cost of the service was staffing. Based on our cost analysis, the average cost per patient seen by the CRRT was £374, with an average cost of £98 pare consultation. This is a significant saving from the average cost per inpatient hospital stay in NHS GGC in 2018–19 of £3602^19^ or £3000 for a COPD-related admission^20^. While it was impossible to capture how many admissions were averted among patients seen during the month analysed, the overall trend of reduced admissions suggests that the service was cost-effective. In a feedback questionnaire completed by 123 General Practitioners (GPs) within the Trust, 82% of respondents stated they would have referred their patients to the hospital had the service not been there and 87% felt that the CRRT was a service that the Trust should retain. The impact on patient wellbeing could be further assessed through patient feedback during an ongoing analysis of the service.

These findings are significant as enhancing community health services is becoming a national priority. Spending on acute services to treat patients with chronic disease is expected to double over the next 15 years^34^. Respiratory conditions are a key contributor to acute admissions, accounting for 850,000 pre-pandemic emergency hospital visits per year, with a yearly cost to the NHS of £9.9 billion^35^. Continuity of care is also important in this often multimorbid group, with integrated respiratory services providing holistic care for patients between the primary and secondary care interface shown to improve patient outcomes^36,37^. The National Institute for Health and Care Excellence (NICE) recommends multidisciplinary hospital-at-home schemes for managing selected patients with exacerbations of COPD^38^ and this study has shown that the NHS GGC CRRT was able to effectively provide this service resulting in a reduction in emergency department attendances for COPD across the Trust, low rates of admission and death amongst CRRT patients, and no nosocomial COVID-19 infections.

The scale of CRRT service delivered in NHS GG&C was made possible by the halting of routine respiratory outpatient services and significant staff redeployment to allow for expanded care in the community. To continue this service beyond the pandemic, when routine services are remobilised, will require an investment of human and financial resources. A cost-effectiveness analysis would aid such service re-design.

We are unaware of any similar initiatives in the UK operating during the COVID-19 pandemic. In addition, few other studies have analysed the effectiveness of multidisciplinary community respiratory care services, but those that have shown generally positive results^8–12^. Our study adds to the volume of evidence supporting the efficacy of multidisciplinary community respiratory care.

There are limitations to our study. As the CRRT was established rapidly in response to the COVID-19 pandemic, there is no previous data for comparison and this was not a randomised control study. As data were collected retrospectively, there is a risk of misclassification bias, and we were unable to capture details such as pathway outcomes in relation to disease severity. It was also not possible to assess reliably the rates of COVID-19 positivity in this patient cohort due to inconsistencies in hospital and community testing regimes. Ongoing prospective and comparative analysis is required to strengthen the validity of our results and to aid future service planning. The method of analysis used for Trust-wide and national data assumes that the impact of COVID-19 is adjusted for by adjusting all EAs. It also assumes that any change in COPD EA trends occurring in RoS between pre- and post-intervention beyond a change in all-cause EAs would also have occurred in GGC had not the CRRT been put in place. It also assumes that there are no other relevant factors that might explain the trend seen in GGC, specifically for COPD EAs after CRRT onset. This methodology can be sensitive to start and end data points and also to outliers. The models were run several different ways, removing the final data point where RoS saw a small rise in COPD EAs, and the conclusion remains.

This study has shown that the NHS GGC CRRT was able to appropriately risk stratify patients, efficiently target resources such as home visits to higher-risk patients, and complement tertiary care by providing support at home associated with a reduction in emergency department attendances among COPD patients. As the COVID-19 pandemic continues, our study suggests the development of multidisciplinary community respiratory service could be more widely implemented with benefits to both patients and healthcare services.

## Supplementary information

REPORTING SUMMARY

Supplementary Table 1

## Data Availability

Anonymised patient-level data were available via email request to the corresponding author.
