# Supplementary material for: Multi-disciplinary community respiratory team management of patients with chronic respiratory illness during the COVID-19 pandemic
Source: NPJ Prim Care Respir Med. 2022 Aug 13;32:26. doi: 10.1038/s41533-022-00290-y (PMC9375196; doi:10.1038/s41533-022-00290-y)
Supplement: Supplementary file 1 — REPORTING SUMMARY [file 41533_2022_290_MOESM1_ESM.pdf]

nature portfolio

Corresponding author(s): Dr Emma Johnson

Last updated by author(s): 27/01/2022

Reporting Summary

Nature Portfolio wishes to improve the reproducibility of the work that we publish. This form provides structure for consistency and transparency in reporting. For further information on Nature Portfolio policies, see our Editorial Policies and the Editorial Policy Checklist.

Statistics

For all statistical analyses, confirm that the following items are present in the figure legend, table legend, main text, or Methods section.

n/a	Confirmed	
☐	☒	The exact sample size (n) for each experimental group/condition, given as a discrete number and unit of measurement
☒	☐	A statement on whether measurements were taken from distinct samples or whether the same sample was measured repeatedly
☐	☒	The statistical test(s) used AND whether they are one- or two-sided<br>*Only common tests should be described solely by name; describe more complex techniques in the Methods section.*
☐	☒	A description of all covariates tested
☐	☒	A description of any assumptions or corrections, such as tests of normality and adjustment for multiple comparisons
☐	☒	A full description of the statistical parameters including central tendency (e.g. means) or other basic estimates (e.g. regression coefficient) AND variation (e.g. standard deviation) or associated estimates of uncertainty (e.g. confidence intervals)
☐	☒	For null hypothesis testing, the test statistic (e.g. F, t, r) with confidence intervals, effect sizes, degrees of freedom and P value noted<br>*Give P values as exact values whenever suitable.*
☒	☐	For Bayesian analysis, information on the choice of priors and Markov chain Monte Carlo settings
☒	☐	For hierarchical and complex designs, identification of the appropriate level for tests and full reporting of outcomes
☒	☐	Estimates of effect sizes (e.g. Cohen's d, Pearson's r), indicating how they were calculated

Our web collection on statistics for biologists contains articles on many of the points above.

Software and code

Policy information about availability of computer code

Data collection	No software was used
Data analysis	All statistical analyses were undertaken on R version 3.4.1

For manuscripts utilizing custom algorithms or software that are central to the research but not yet described in published literature, software must be made available to editors and reviewers. We strongly encourage code deposition in a community repository (e.g. GitHub). See the Nature Portfolio guidelines for submitting code & software for further information.

Data

Policy information about availability of data

All manuscripts must include a data availability statement. This statement should provide the following information, where applicable:

- Accession codes, unique identifiers, or web links for publicly available datasets
- A description of any restrictions on data availability
- For clinical datasets or third party data, please ensure that the statement adheres to our policy

Anonymised patient level data are available from the authors on request.

Field-specific reporting

Please select the one below that is the best fit for your research. If you are not sure, read the appropriate sections before making your selection.

☐ Life sciences ☒ Behavioural & social sciences ☐ Ecological, evolutionary & environmental sciences

For a reference copy of the document with all sections, see nature.com/documents/nr-reporting-summary-flat.pdf

Behavioural & social sciences study design

All studies must disclose on these points even when the disclosure is negative.

Study description	This was a population-based, retrospective, observational study.
Research sample	Data was collected from patient electronic records of all patients referred to the CRRT during the month of May 2020. This totalled 516 patients. All were resident within the NHS GGC health board area.
Sampling strategy	No sample size calculation was performed. Data was collected for all patients seen by the service during the month of May 2020. There were no exclusion criteria and no changes were made to the service after May 2020 so we believe the sample is representative of all patients seen during the months in which the CRRT was active.
Data collection	Data on reason for referral, triage category, primary respiratory diagnosis and patient demographics were collected from the original referral documentation to the CRRT. Information on number and type of consultations was collected from the CRRT electronic consultation notes. Data on admissions and deaths within 28 days of referral to CRRT were collected from hospital admission records and, where further information was necessary, from inpatient notes and discharge letters viewable in the electronic patient record. The six teams provided total numbers of staff involved, percentage of their time committed to the CRRT, and number of staff working per day. These were used in conjunction with payroll details to calculate the cost of staffing the service. Monthly emergency attendance (EA) data for all causes and COPD only (COPD EA) from January 2018 to May 2021 were obtained for NHS GGC and the rest of Scotland, with the exception of Fife, Lothian and Tayside health boards, from Public Health Scotland. COPD EAs are defined as those coded as ICD-10 J40-J44 .
Timing	Data was collected between September and December 2020.
Data exclusions	There were no exclusion criteria.
Non-participation	As this was a retrospective study, no patient dropped out.
Randomization	n/a

Reporting for specific materials, systems and methods

We require information from authors about some types of materials, experimental systems and methods used in many studies. Here, indicate whether each material, system or method listed is relevant to your study. If you are not sure if a list item applies to your research, read the appropriate section before selecting a response.

Materials & experimental systems

n/a	Involved in the study	
☒	☐	Antibodies
☒	☐	Eukaryotic cell lines
☒	☐	Palaeontology and archaeology
☒	☐	Animals and other organisms
☒	☐	Human research participants
☐	☒	Clinical data
☒	☐	Dual use research of concern

Methods

n/a	Involved in the study	
☒	☐	ChIP-seq
☒	☐	Flow cytometry
☒	☐	MRI-based neuroimaging

Clinical data

Policy information about clinical studies

All manuscripts should comply with the ICMJE guidelines for publication of clinical research and a completed CONSORT checklist must be included with all submissions.

Clinical trial registration	n/a
Study protocol	this was not a clinical trial

Data collection	Data was collected from patient electronic records of all patients referred to the CRRT during the month of May 2020. Data on reason for referral, triage category, primary respiratory diagnosis and patient demographics were collected from the original referral documentation to the CRRT. Information on number and type of consultations was collected from the CRRT electronic consultation notes. Data on admissions and deaths within 28 days of referral to CRRT were collected from hospital admission records and, where further information was necessary, from inpatient notes and discharge letters viewable in the electronic patient record.
Outcomes	The primary aims were to determine 1) whether the triage system was effective in identifying high risk patients; 2) how extensively remote consultations were used and face-to-face consultations targeted towards patients most in need; and 3) whether CRRT input affected rates of emergency department attendances (EAs), hospital admission and death among patients at 28 days after initial consultation.
