# Supplementary material for: Multi-disciplinary community respiratory team management of patients with chronic respiratory illness during the COVID-19 pandemic
Source: NPJ Prim Care Respir Med. 2022 Aug 13;32:26. doi: 10.1038/s41533-022-00290-y (PMC9375196; doi:10.1038/s41533-022-00290-y)
Supplement: Supplementary file 2 — Supplementary Table 1 [file 41533_2022_290_MOESM2_ESM.pdf]

Supplementary Table 1: CRRT Patient Primary Respiratory Diagnosis, Triage Category and Outcomes

Primary Respiratory Diagnosis	Pathway	n	ED Attendance		Hospital Admission		Death	
			n	% of Pathway	n	% of Pathway	n	% of Pathway
COPD	RED	77	16	21%	16	21%	3	4%
	AMBER	158	19	12%	19	12%	4	3%
	GREEN	88	8	9%	8	9%	1	1%
	BLUE	13	2	15%	2	15%	3	23%
	TOTAL	**336**	**45**	**13%**	**45**	**13%**	**11**	**3%**
ASTHMA	RED	6	1	17%	1	17%	0	-
	AMBER	30	4	13%	2	7%	0	-
	GREEN	27	1	4%	1	4%	0	-
	BLUE	0	0	-	0	-	0	-
	TOTAL	**63**	**6**	**10%**	**4**	**6%**	**0**	**-**
ILD	RED	3	0	-	0	-	0	-
	AMBER	19	1	5%	0	-	0	-
	GREEN	9	1	11%	1	11%	0	-
	BLUE	5	1	20%	0	-	0	-
	TOTAL	**36**	**3**	**8%**	**1**	**3%**	**0**	**-**
ASTHMA/COPD OVERLAP	RED	7	1	14%	1	14%	0	-
	AMBER	20	2	10%	3	15%	0	5%
	GREEN	5	0	-	0	-	0	-
	BLUE	2	0	-	0	-	1	50%
	TOTAL	**34**	**3**	**9%**	**4**	**12%**	**1**	**3%**
COVID	RED	0	0	-	0	-	0	-
	AMBER	1	0	-	0	-	0	-
	GREEN	1	0	-	0	-	0	-
	BLUE	9	2	22%	2	22%	3	44%
	TOTAL	**11**	**2**	**18%**	**2**	**18%**	**3**	**27%**
BRONCHIECTASIS	RED	4	1	25%	2	50%	1	25%
	AMBER	5	2	40%	2	40%	0	-
	GREEN	1	0	-	0	-	0	-
	BLUE	0	0	-	0	-	0	-
	TOTAL	**10**	**3**	**30%**	**4**	**40%**	**1**	**10%**
EOL	RED	0	0	-	0	-	0	-
	AMBER	0	0	-	0	-	0	-
	GREEN	1	0	-	0	-	0	-
	BLUE	8	1	13%	1	13%	5	63%
	TOTAL	**9**	**1**	**11%**	**1**	**11%**	**5**	**56%**
OTHER/UNNOWN	RED	2	2	100%	1	50%	0	-
	AMBER	2	0	-	0	-	0	-
	GREEN	10	1	10%	1	10%	0	-
	BLUE	3	0	-	0	-	2	67%
	TOTAL	**17**	**3**	**18%**	**2**	**12%**	**2**	**12%**

"-" denotes nil in category
